# Sample Size Determination for Comparing Slopes in Cluster Randomized Trials With Longitudinal Measurements

**DOI:** 10.1002/sim.70576

**Published:** 2026-05-04

**Authors:** Jijia Wang, Song Zhang, Chul Ahn

**Affiliations:** ^1^ Department of Applied Clinical Research UT Southwestern Medical Center Dallas Texas USA; ^2^ Peter O'Donnell School of Public Health UT Southwestern Medical Center Dallas Texas USA

**Keywords:** cluster randomized trial with longitudinal measurements, generalized estimating equation, sample size, slope comparison

## Abstract

In cluster randomized trials with longitudinal measurements (CRTLMs), clusters of subjects, rather than individual subjects, are randomly assigned to either control or intervention groups. Measurements are collected from these subjects repeatedly at prespecified times until the end of the study. In clinical research, the focus is typically on investigating trends or progress over time to evaluate the effectiveness of a new treatment or track disease progression, rather than solely analyzing mean values or endpoint measurements. For comparing slopes between two groups, we have derived closed‐form sample size formulas based on the generalized estimating equation (GEE) approach under independence working correlation. Our proposed method is highly flexible, allowing for the incorporation of unbalanced randomization, arbitrary correlation structures, various missing data scenarios through observational probabilities and missing patterns, and variability in cluster sizes. This flexibility provides a practical and robust sample size solution for CRTLMs. Simulation studies demonstrate that the proposed method performs well, maintaining empirical power and type I error rate close to their nominal values. Additionally, we illustrate the application of our method using a real clinical trial, showcasing its practical utility in real‐world implementation.

**Abbreviations**
CRTLMs: cluster randomized trial with longitudinal measurements
GEE: generalized estimating equation

## 1 Introduction

A cluster randomized trial with longitudinal measurements (CRTLMs) is a specific type of randomized trial widely used in medical, educational, and social sciences research [1, 2]. Instead of randomizing individual subjects, clusters of subjects (e.g., schools, communities, hospitals, or other units) are randomly assigned to different treatment groups. The outcome for each subject is measured at multiple prespecified time points. This longitudinal aspect allows researchers to understand how the treatment effect evolves, persists, or diminishes over time. The CRTLM design offers several advantages [2, 3]. Randomization at the cluster level can enhance the feasibility and logistical convenience of the trial, particularly when individual randomization is impractical or would lead to contamination. In addition, CRTLMs are naturally compatible with interventions administered at the group level, such as educational programs or public health initiatives. Furthermore, CRTLMs provide enriched data by capturing the trajectory of measurements over time, enabling a more granular evaluation of the treatment effect. Finally, the repeated measurements can lead to greater statistical power compared to trials with a single outcome measurement per subject.

On the other hand, the CRTLM design presents its own challenges. One major challenge is the complexity of correlations, which must be appropriately accounted for in both study design and data analysis [4, 5, 6]. These correlations are multifaceted. They include within‐subject (longitudinal) correlation, which is the correlation between measurements taken over time from the same subject, and between‐subject correlations, which encompass between‐subject within‐period correlation (also known as the intra‐cluster correlation, ICC) and between‐subject between‐period correlation, which is the correlation between nonconcurrent measurements from different subjects within the same cluster. Ignoring these correlations can lead to biased inferences and inappropriate trial design. Another potential challenge in CRTLMs is missing data, especially in studies with prolonged follow‐up. Subjects may drop out, miss scheduled assessments, or have incomplete data for random reasons. Nonetheless, by addressing these challenges proactively, researchers can leverage CRTLMs to enhance the robustness and reliability of their findings on treatment effects.

Researchers typically design CRTLMs based on two types of inference: comparing the time‐averaged mean between treatment groups or comparing the trends of outcomes over time. Comparing trends (slopes) rather than time‐averaged means offers a deeper understanding of how treatment effects evolve over time. It can reveal how quickly patients respond to interventions and whether the benefits are sustained. Such insights are crucial for designing effective treatment strategies and making informed decisions in clinical practice. For instance, consider a CRTLM aimed at assessing the impact of an intervention on reducing the severity of patients' depression symptoms. Clinics are randomly assigned to either the intervention or control group, and each subject is repeatedly measured for depression severity over two years. The comparison of slopes allows researchers to evaluate not only the immediate effects of the intervention but also how these effects progress over time. Researchers can determine whether the intervention results in a more rapid or sustained improvement in symptoms compared to the control.

In this study, we investigate sample size calculation for CRTLMs aimed at comparing the slopes between two groups. Multiple sample size calculation methods have been developed based on two commonly used analytical approaches: the generalized linear mixed model (GLMM) and the generalized estimating equation (GEE) [5, 7, 8]. For example, within the GLMM framework, Heo et al. [9] developed a sample size calculation method that utilizes the maximum likelihood estimate to assess the intervention‐by‐time interaction (difference in slopes) in CRTLMs. This method assumes balanced randomization as well as constant longitudinal and between‐subject correlations. The authors acknowledged that practical scenarios often involve variability in cluster size and the number of measurements per subject, which is more common than exceptional. To address these limitations, Heo et al. [10] proposed a closed‐form approximate sample size calculation that accounts for anticipated attrition rates. They modified an existing sample size formula to accommodate varying attrition rates, assuming that the distribution of attrition times is either uniform over the study period or linearly increasing. This adaptation allows for a more realistic and practical determination of sample sizes in the presence of subject dropout. Murray et al. [11] developed a method for determining detectable effect size and sample size based on expected mean square errors using random coefficients analysis for nested cohort designs. However, it did not address the issue of attrition. Within the GEE framework, Liu et al. [12] derived a sample size formula for comparing two slopes in CRTLMs. It assumes specific correlation structures, such as proportional shrinkage compound symmetry and autoregressive structures, and accounts for variability in cluster size based on detailed distribution information. However, it did not consider missing data, which is a common issue in longitudinal studies. Additionally, this study did not include simulation studies to evaluate the empirical performance of the proposed method. We propose a flexible sample size calculation approach using GEE for CRTLMs, where the primary interest lies in the comparison of two slopes. The proposed method offers closed‐form solutions that accommodate unbalanced randomization, arbitrary correlation structures, various missing data scenarios, and randomly varying cluster sizes. We have conducted extensive simulations across various design configurations to assess its performance in practical scenarios. Finally, we address a common issue in cluster randomized trials by proposing practical adjustment methods to ensure the validity of statistical inference when the number of clusters is small. This issue has been widely discussed in literature [13, 14, 15].

This paper is organized as follows. Section 2 introduces the GEE model and derives a closed‐form sample size formula. This formula accommodates longitudinal measurements, unbalanced randomization, arbitrary correlation structures, various missing data scenarios, and varying cluster sizes. Section 3 presents extensive simulation studies to evaluate the performance of the proposed method. We investigate how different design parameters affect sample size requirements and provide practical recommendations based on these findings. Section 4 illustrates the application of the proposed sample size calculation method through a detailed example. Section 5 concludes with a discussion.

## 2 Sample Size Calculation Using GEE

Consider a CRTLM in which n clusters are randomly assigned to either the control or intervention group. Let $u_{i}=0/1$ indicate that cluster i (i=1,…,n) is randomized to the control/intervention group. Cluster sizes are denoted by $J_{i}$, which may vary across clusters. Each subject provides T repeated measurements. Let $y_{ijr}$ denote the continuous outcome collected from subject j ($j=1,\ldots ,J_{i}$) within cluster i (i=1,⋯,n) at time $t_{r}$ (r=1,…,T). Under the GEE framework, we need to specify models for the first two moments of $y_{ijr}$. We model the marginal mean of $y_{ijr}$ by:

(1) $$E(y_{ijr})=\beta_{1}+\beta_{2}u_{i}+\beta_{3}t_{r}+\beta_{4}u_{i}t_{r}.$$

Here, $\beta_{1}$ and $\beta_{3}$ represent the intercept and slope of the control group, while $\beta_{2}$ and $\beta_{4}$ represent the differences in intercept and slope between the two treatment groups, respectively. In CRTLMs, we are usually interested in testing the null hypothesis $H_{0}:\beta_{4}=0$ to determine whether there is a significant difference in slope between the two treatment groups. The variance is assumed to be $\mathrm{Var}\left(y_{ijr}\right)=\sigma^{2}$. The within‐subject (longitudinal) correlation matrix for $y_{ij}={\left(y_{ij1},\ldots ,y_{ijT}\right)}^{\prime }$ is

$$\Omega =\left(\begin{array}{cccc} 1 & \omega_{12} & \cdots & \omega_{1T}\\ \omega_{21} & 1 & \cdots & \omega_{2T}\\ \vdots & \vdots & \ddots & \vdots \\ \omega_{T1} & \omega_{T2} & \cdots & 1 \end{array}\right),$$

where $\omega_{rr^{\prime }}=\omega_{r^{\prime }r}=\text{Corr}(y_{ijr},y_{ijr^{\prime }})$ for $r\neq r^{\prime }$. Within each cluster, the between‐subject correlation matrix for $y_{ij}$ and $y_{ij^{\prime }}$ ($j\neq j^{\prime }$) is denoted by $\Phi ={\left\{\phi_{rr^{\prime }}\right\}}_{T\times T}$, where the diagonal elements $\phi_{rr}=\text{Corr}(y_{ijr},y_{ij^{\prime }r})$ represent the traditional intra‐cluster correlation (ICC) at time $t_{r}$ (r=1,…,T), and off‐diagonal elements $\phi_{rr^{\prime }}=\phi_{r^{\prime }r}=\text{Corr}(y_{ijr},y_{ij^{\prime }r^{\prime }})$ quantify the correlation between nonconcurrent measurements from different subjects within the same cluster, which we refer to as the between‐subject between‐period correlation. Define $y_{i}={\left(y_{i1}^{\prime },\ldots ,y_{iJ_{i}}^{\prime }\right)}^{\prime }$ as the collection of outcome measurements from the ith cluster. Its correlation matrix is fully specified by Ω and Φ:

$$R=I_{J_{i}}⊗\left(\Omega -\Phi \right)+\left(1_{J_{i}}1_{J_{i}}^{\prime }\right)⊗\Phi ,$$

where ⊗ denotes the Kronecker product, $I_{J_{i}}$ is a $J_{i}\times J_{i}$ identity matrix, and $1_{J_{i}}$ is a vector of length $J_{i}$ with all elements equal to 1. Finally, we assume that measurements from different clusters are independent. This completes the GEE model specification.

### 2.1 Sample Size Under Complete Observations

We begin by deriving the sample size formula for CRTLMs with complete observations. To simplify derivation, let $p=E(u_{i})$ denote the proportion of clusters randomized to the intervention group. Then Model (1) can be rewritten as:

(2) $$E(y_{ijr})=b_{1}+b_{2}(u_{i}-p)+b_{3}t_{r}+b_{4}(u_{i}-p)t_{r},$$

where $b_{1}=\beta_{1}+\beta_{2}p$, $b_{2}=\beta_{2}$, $b_{3}=\beta_{3}+\beta_{4}p$, and $b_{4}=\beta_{4}$. Define $b={\left(b_{1},b_{2},b_{3},b_{4}\right)}^{\prime }$, $Z_{ijr}=\left(1,u_{i}-p,t_{r},(u_{i}-p)t_{r}\right)$, and $Z_{ij}=\left(1_{T},(u_{i}-p)1_{T},t,(u_{i}-p)t\right)\equiv Z_{i}$ with $t={\left(t_{1},\cdots \;,t_{T}\right)}^{\prime }$. Using the independent working correlation structure [16, 17], the GEE estimator $\hat{b}={\left({\hat{b}}_{1},{\hat{b}}_{2},{\hat{b}}_{3},{\hat{b}}_{4}\right)}^{\prime }$ can be solved from equation $S_{n}(b)=0$, where

(3) $$S_{n}(b)=n^{-1/2}\overset{n}{\underset{i=1}{\sum }}\overset{J_{i}}{\underset{j=1}{\sum }}Z_{i}^{\prime }(y_{ij}-Z_{i}b).$$

Specifically,

(4) $$\begin{array}{ll} \hat{b} & ={\left(\overset{n}{\underset{i=1}{\sum }}\overset{J_{i}}{\underset{j=1}{\sum }}Z_{i}^{\prime }Z_{i}\right)}^{-1}\left(\overset{n}{\underset{i=1}{\sum }}\overset{J_{i}}{\underset{j=1}{\sum }}Z_{i}^{\prime }y_{ij}\right)\\ & ={\left(\overset{n}{\underset{i=1}{\sum }}\overset{J_{i}}{\underset{j=1}{\sum }}\overset{T}{\underset{r=1}{\sum }}Z_{ijr}^{\prime }Z_{ijr}\right)}^{-1}\left(\overset{n}{\underset{i=1}{\sum }}\overset{J_{i}}{\underset{j=1}{\sum }}\overset{T}{\underset{r=1}{\sum }}Z_{ijr}^{\prime }y_{ijr}\right). \end{array}$$

Liang and Zeger [18] demonstrated that $\sqrt{n}(\hat{b}-b)$ approximately follows a normal distribution with a mean of zero and a covariance consistently estimated by $\sum_{n}=A_{n}^{-1}V_{n}A_{n}^{-1}$, where

$$\begin{array}{ll} A_{n} & =n^{-1}\overset{n}{\underset{i=1}{\sum }}\overset{J_{i}}{\underset{j=1}{\sum }}\overset{T}{\underset{r=1}{\sum }}Z_{ijr}^{\prime }Z_{ijr}\\ & =n^{-1}\overset{n}{\underset{i=1}{\sum }}\overset{J_{i}}{\underset{j=1}{\sum }}\overset{T}{\underset{r=1}{\sum }}\\ & \;\left[\begin{array}{cccc} 1 & u_{i}-p & t_{r} & \left(u_{i}-p\right)t_{r}\\ u_{i}-p & {\left(u_{i}-p\right)}^{2} & \left(u_{i}-p\right)t_{r} & {\left(u_{i}-p\right)}^{2}t_{r}\\ t_{r} & \left(u_{i}-p\right)t_{r} & t_{r}^{2} & \left(u_{i}-p\right)t_{r}^{2}\\ \left(u_{i}-p\right)t_{r} & {\left(u_{i}-p\right)}^{2}t_{r} & \left(u_{i}-p\right)t_{r}^{2} & {\left(u_{i}-p\right)}^{2}t_{r}^{2} \end{array}\right] \end{array}$$

and 


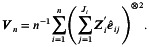


Here, ${\hat{e}}_{ij}=y_{ij}-Z_{i}\hat{b}$ is the vector of residuals, and $c^{⊗2}=cc^{\prime }$ for a vector c. Let ${\hat{\sigma }}_{4}^{2}$ denote the (4, 4)th element of $\sum_{n}$. We will reject the null hypothesis $H_{0}:\beta_{4}=0$ if $\left|\sqrt{n}{\hat{\beta }}_{4}/{\hat{\sigma }}_{4}\right|>z_{1-\alpha /2}$, where α is the significance level and $z_{1-\alpha /2}$ is the 100(1−α/2)th percentile of the standard normal distribution.

Define $A=\lim_{n\to \infty }A_{n}$ and $V=\lim_{n\to \infty }V_{n}$. Suppose the cluster size $J_{i}$ follows a specific distribution with mean J and variance $\sigma_{J}^{2}$. Then we have

$$A=JT\left[\begin{array}{cccc} 1 & 0 & \mu_{1} & 0\\ 0 & v_{p}^{2} & 0 & v_{p}^{2}\mu_{1}\\ \mu_{1} & 0 & \mu_{2} & 0\\ 0 & v_{p}^{2}\mu_{1} & 0 & v_{p}^{2}\mu_{2} \end{array}\right]$$

and

$$V=\sigma^{2}J^{2}\left[\begin{array}{cccc} 1_{T}^{\prime }Q1_{T} & 0 & 1_{T}^{\prime }Qt & 0\\ 0 & v_{p}^{2}1_{T}^{\prime }Q1_{T} & 0 & v_{p}^{2}1_{T}^{\prime }Qt\\ t^{\prime }Q1_{T} & 0 & t^{\prime }Qt & 0\\ 0 & v_{p}^{2}t^{\prime }Q1_{T} & 0 & v_{p}^{2}t^{\prime }Qt \end{array}\right],$$

where $\mu_{1}=\sum_{r=1}^{T}t_{r}/T$, $\mu_{2}=\sum_{r=1}^{T}t_{r}^{2}/T$, $v_{p}^{2}=p(1-p)$, and $Q=\frac{1}{J}\Omega +(CV_{J}^{2}+1-\frac{1}{J})\Phi$ with $CV_{J}=\sigma_{J}/J$ being the coefficient of variation for cluster size. Define $\sum =\lim_{n\to \infty }\sum_{n}=A^{-1}VA^{-1}$. Let $\sigma_{4}^{2}$ denote the (4,4)th element in ∑. Then

(5) $$\sigma_{4}^{2}=\frac{\sigma^{2}}{T^{2}v_{p}^{2}v_{t}^{4}}{\left(\mu_{1}1_{T}-t\right)}^{\prime }Q\left(\mu_{1}1_{T}-t\right),$$

where $v_{t}^{2}=\mu_{2}-\mu_{1}^{2}$. The details of the derivation are presented in Appendix A. Given the true value of the intervention effect $\beta_{40}$, to achieve a statistical power of 1−γ with a two‐sided type I error rate of α, the required sample size for a CRTLM with complete observations is

(6) $$n_{complete}=\frac{{\left(z_{1-\alpha /2}+z_{1-\gamma }\right)}^{2}\sigma^{2}{\left(\mu_{1}1_{T}-t\right)}^{\prime }Q\left(\mu_{1}1_{T}-t\right)}{\beta_{40}^{2}T^{2}v_{p}^{2}v_{t}^{4}}.$$

It is noteworthy that $n_{complete}$ is identical to Formula (42) in Liu et al. [12] under global homogeneous correlation and to Formula (15) in Heo et al. [9] based on maximum likelihood estimation. Murray et al. [11] considered additional sources of variation, including variation in slopes and intercepts at both the cluster and individual levels. Their specification effectively estimates the correlation by accounting for the additional variance attributed to the cluster, beyond the variance explained by individual‐level factors. In contrast, our study incorporates correlation settings through the specification of matrices Ω and Φ within Q in Formula (6).

### 2.2 Sample Size Under Incomplete Observations

For CRTLMs with incomplete observations, let $m_{ijr}=0/1$ indicate that $y_{ijr}$ is missing/observed. Then the generalized $A_{n}$ and $V_{n}$ under incomplete observations are

(7) $$\begin{array}{cc} A_{n} & =n^{-1}\overset{n}{\underset{i=1}{\sum }}\overset{J_{i}}{\underset{j=1}{\sum }}\overset{T}{\underset{r=1}{\sum }}Z_{ijr}^{\prime }Z_{ijr}m_{ijr}\\ & =n^{-1}\overset{n}{\underset{i=1}{\sum }}\overset{J_{i}}{\underset{j=1}{\sum }}\overset{T}{\underset{r=1}{\sum }}m_{ijr}\\ & \;\left[\begin{array}{cccc} 1 & u_{i}-p & t_{r} & \left(u_{i}-p\right)t_{r}\\ u_{i}-p & {\left(u_{i}-p\right)}^{2} & \left(u_{i}-p\right)t_{r} & {\left(u_{i}-p\right)}^{2}t_{r}\\ t_{r} & \left(u_{i}-p\right)t_{r} & t_{r}^{2} & \left(u_{i}-p\right)t_{r}^{2}\\ \left(u_{i}-p\right)t_{r} & {\left(u_{i}-p\right)}^{2}t_{r} & \left(u_{i}-p\right)t_{r}^{2} & {\left(u_{i}-p\right)}^{2}t_{r}^{2} \end{array}\right] \end{array}$$

and 

(8)

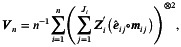


where $m_{ij}={\left(m_{ij1},\ldots ,m_{ijT}\right)}^{\prime }$ and ∘ denotes the Hadamard product. ${\hat{\sigma }}_{4}^{2}$ can be consistently estimated by the (4, 4)th element of $\sum_{n}=A_{n}^{-1}V_{n}A_{n}^{-1}$. We assume the occurrence of missing data to be time‐dependent with marginal observational probability $\delta_{r}=P(m_{ijr}=1)$ for r=1,⋯,T. Usually, the occurrence of missing data increases over time, so $\delta_{1}\geq \ldots \geq \delta_{T}$. We also define $\delta_{rr^{\prime }}=P(m_{ijr}m_{ijr^{\prime }}=1)$, the joint probability of a subject providing observations at times $t_{r}$ and $t_{r^{\prime }}$ ($r\neq r^{\prime }$) simultaneously, which allows us to account for different missing patterns. Given the same set of marginal probabilities $\delta_{r}$'s, different missing patterns lead to different values of $\delta_{rr^{\prime }}$'s. For example,

1. under the independent missing (IM) pattern, where missing data occur independently over time, we have $\delta_{rr^{\prime }}=\delta_{r}\delta_{r^{\prime }}$ for $r\neq r^{\prime }$;
2. under the monotone missing (MM) pattern, where a subject having missing data at time $t_{r}$ misses all subsequent observations, we have $\delta_{rr^{\prime }}=\delta_{r^{\prime }}$ for $r<r^{\prime }$; and
3. under mixed missing (MIX) pattern, the occurrence of missing data follows a mixture of IM and MM with weights w and 1−w, respectively. Thus, $\delta_{rr^{\prime }}=w\delta_{r}\delta_{r^{\prime }}+(1-w)\delta_{r^{\prime }}$.

Under the assumption of missing completely at random, let $A=\lim_{n\to \infty }A_{n}$ and $V=\lim_{n\to \infty }V_{n}$, we have

$$A=J\mu_{m0}\left[\begin{array}{cccc} 1 & 0 & \mu_{m1} & 0\\ 0 & v_{p}^{2} & 0 & v_{p}^{2}\mu_{m1}\\ \mu_{m1} & 0 & \mu_{m2} & 0\\ 0 & v_{p}^{2}\mu_{m1} & 0 & v_{p}^{2}\mu_{m2} \end{array}\right]$$

and

$$V=\sigma^{2}J^{2}\left[\begin{array}{cccc} 1_{T}^{\prime }Q_{m}1_{T} & 0 & 1_{T}^{\prime }Q_{m}t & 0\\ 0 & v_{p}^{2}1_{T}^{\prime }Q_{m}1_{T} & 0 & v_{p}^{2}1_{T}^{\prime }Q_{m}t\\ t^{\prime }Q_{m}1_{T} & 0 & t^{\prime }Q_{m}t & 0\\ 0 & v_{p}^{2}t^{\prime }Q_{m}1_{T} & 0 & v_{p}^{2}t^{\prime }Q_{m}t \end{array}\right],$$

where $\mu_{m0}=\sum_{r=1}^{T}\delta_{r}$, $\mu_{m1}=\sum_{r=1}^{T}\delta_{r}t_{r}/\mu_{m0}$, $\mu_{m2}=\sum_{r=1}^{T}\delta_{r}t_{r}^{2}/\mu_{m0}$, and $Q_{m}=\frac{1}{J}\Omega \circ \Delta +\left(CV_{J}^{2}+1-\frac{1}{J})\text{diag}(\delta \right)\Phi \text{diag}(\delta )$. Here diag(δ) is a diagonal matrix with diagonal elements being $\delta ={\left(\delta_{1},\ldots ,\delta_{T}\right)}^{\prime }$ and Δ is a matrix with the (r,r)th element being $\delta_{r}$ and the $\left(r,r^{\prime }\right)$th element being $\delta_{rr^{\prime }}$ ($r\neq r^{\prime }$). Consequently, the (4,4)th element in $\sum =A^{-1}VA^{-1}$ is

(9) $$\sigma_{4}^{2}=\frac{\sigma^{2}}{\mu_{m0}^{2}v_{p}^{2}v_{mt}^{4}}{\left(\mu_{m1}1_{T}-t\right)}^{\prime }Q_{m}\left(\mu_{m1}1_{T}-t\right),$$

where $v_{mt}^{2}=\mu_{m2}-\mu_{m1}^{2}$. The details of the derivation are presented in Appendix A. Given the true value of the intervention effect $\beta_{40}$, to achieve a statistical power of 1−γ with a two‐sided type I error rate of α, the required sample size for a CRTLM with incomplete observations is

(10) $$n=\frac{{\left(z_{1-\alpha /2}+z_{1-\gamma }\right)}^{2}\sigma^{2}{\left(\mu_{m1}1_{T}-t\right)}^{\prime }Q_{m}\left(\mu_{m1}1_{T}-t\right)}{\beta_{40}^{2}\mu_{m0}^{2}v_{p}^{2}v_{mt}^{4}}.$$

On the other hand, given the number of clusters (n), the statistical power of the study can be calculated using

(11) $$P\left(Z<\frac{\sqrt{n}|\beta_{40}|\mu_{m0}v_{p}v_{mt}^{2}}{\sigma \sqrt{{\left(\mu_{m1}1_{T}-t\right)}^{\prime }Q_{m}\left(\mu_{m1}1_{T}-t\right)}}-z_{1-\alpha /2}\right),$$

where Z is a random variable following the standard normal distribution.

The closed‐form sample size Formula (10) for CRTLMs indicates that several key parameters need to be specified: the true intervention effect ($\beta_{40}$) measured as slope difference between the intervention and control groups; the variance of the measurements ($\sigma^{2}$); the proportion of randomization (p); the first two moments of randomly varying cluster sizes (J and $\sigma_{J}^{2}$); the longitudinal correlation matrix (Ω) and the between‐subject correlation matrix (Φ); the time points of measurements (t); the observational probabilities ($\delta_{r}$'s and $\delta_{rr^{\prime }}$'s); and the desired levels of type I error (α) and power (1−γ). In addition, we have several observations according to this formula. First, the model parameters $\left(\beta_{1},\beta_{2},\beta_{3}\right)$ do not affect the sample size. Second, an increase in the intervention effect $\beta_{40}$ results in a decrease in the required sample size and a balanced design with p=0.5 yields the smallest sample size given all the other parameters. Third, an increase in the variability of cluster size $CV_{J}$ leads to a larger sample size. Fourth, given the same marginal observational probabilities ($\delta_{r}$'s) and nonnegative longitudinal correlations ($\omega_{rr^{\prime }}$'s), the MM pattern always leads to an equal or larger sample size than the IM pattern. This can be easily proved by rewriting the Formula (10) as

$$\begin{array}{ll} n & =\frac{\begin{array}{cc} & {\left(z_{1-\alpha /2}+z_{1-\gamma }\right)}^{2}\sigma^{2}\sum_{r=1}^{T}\sum_{r^{\prime }=1}^{T}\left(\mu_{m1}-t_{r}\right)\left(\mu_{m1}-t_{r}^{\prime }\right)\\ & \left[\frac{1}{J}\omega_{rr^{\prime }}\delta_{rr^{\prime }}+\left(CV_{J}^{2}+1-\frac{1}{J}\right)\phi_{rr^{\prime }}\delta_{r}\delta_{r}^{\prime }\right] \end{array}}{\beta_{40}^{2}\mu_{m0}^{2}v_{p}^{2}v_{mt}^{4}}, \end{array}$$

and using the fact that $\delta_{rr^{\prime }}=\delta_{r^{\prime }}$ ($r<r^{\prime }$) under MM is equal or larger than $\delta_{rr^{\prime }}=\delta_{r}\delta_{r^{\prime }}$ under IM.

If the cluster size is fixed in a CRTLMs study, the sample size calculation Formula (10) can be simplified to

$$n_{fixed}=\frac{{\left(z_{1-\alpha /2}+z_{1-\gamma }\right)}^{2}\sigma^{2}{\left(\mu_{m1}1_{T}-t\right)}^{\prime }Q_{\text{fixed}}\left(\mu_{m1}1_{T}-t\right)}{\beta_{40}^{2}\mu_{m0}^{2}v_{p}^{2}v_{mt}^{4}},$$

where $Q_{\text{fixed}}=\frac{1}{J}\Omega \circ \Delta +\left(1-\frac{1}{J}\right)\text{diag}(\delta )\Phi \text{diag}(\delta )$. In the special case where the cluster size J=1, the design reduces to an individual longitudinal study. The number of subjects required can be calculated by

$$n_{ind}=\frac{{\left(z_{1-\alpha /2}+z_{1-\gamma }\right)}^{2}\sigma^{2}{\left(\mu_{m1}1_{T}-t\right)}^{\prime }\left(\Omega \circ \Delta \right)\left(\mu_{m1}1_{T}-t\right)}{\beta_{40}^{2}\mu_{m0}^{2}v_{p}^{2}v_{mt}^{4}}.$$

It is noteworthy that $n_{ind}$ is identical to the sample size Formula (9) in Jung and Ahn [19].

## 3 Simulation Studies

We conducted simulation studies to evaluate the performance of the proposed sample size method across various design configurations. The parameter settings below were determined based on practical considerations and the literature [20]. Suppose we are planning a CRTLM with balanced randomization (p=0.5). We set the ancillary parameters $\left(\beta_{1},\beta_{2},\beta_{3})=(0,0,0\right)$, which are not involved in the sample size Formula (10). We also set variance $\sigma^{2}=1$. For a two‐sided significance level of α=0.05 and a desired power of 1−γ=0.8, we prespecified the number of time points T as 3, 6, and 12, respectively. For each T, we set time $t_{r}=r-1$ for r=1,⋯,T, and an intervention effect $\beta_{4}=0.2/(T-1)$. For example, when T=3, we have $t={\left(0,1,2\right)}^{\prime }$ and $\beta_{4}=0.1$. In addition, we assumed cluster size $J_{i}$ to follow different discrete uniform (DU) distributions with no, moderate, and large availability, denoted by ${\text{DU}}_{0}$, ${\text{DU}}_{1}$, and ${\text{DU}}_{2}$, respectively. The simulation settings are summarized in Table 1.

**TABLE 1:** Simulation settings.

Parameter	Notation	Value
Randomization ratio	p	0.5
Ancillary parameters	$\left(\beta_{1},\beta_{2},\beta_{3}\right)$	(0, 0, 0)
Variance	$\sigma^{2}$	1
Significance level	α	0.05
Power	1−γ	0.8
Number of time points	T	3, 6, 12
Mean cluster size	J	5, 15, 30
Discrete uniform distribution for cluster size	$DU_{0}$	J=5: DU[5, 5], J=15: DU[15, 15], J=30: DU[30, 30]
	$DU_{1}$	J=5: DU[3, 7], J=15: DU[10, 20], J=30: DU[15, 45]
	$DU_{2}$	J=5: DU[1, 9], J=15: DU[5, 25], J=30: DU[5, 55]
Correlation matrix structure		CS, AR(1)
Combinations of correlations	$R=\left\{\rho_{1},\rho_{2},\rho_{3}\right\}$	$R_{1}=\{0.30,0.05,0.025\}$
		$R_{2}=\{0.30,0.03,0.015\}$
		$R_{3}=\{0.15,0.05,0.025\}$
		$R_{4}=\{0.15,0.03,0.015\}$
Missing data patterns		IM, MM, MIX
Observational probabilities	δ	$\delta_{1}=(1.00,1.00,1.00,1.00,1.00,1.00)$
		$\delta_{2}=(1.00,0.97,0.94,0.90,0.75,0.60)$
		$\delta_{3}=(1.00,0.92,0.84,0.76,0.68,0.60)$
		$\delta_{4}=(1.00,0.85,0.70,0.66,0.63,0.60)$

Different correlation structures were explored. For the longitudinal correlation matrix (Ω), we investigated the compound symmetry (CS) and AR(1) structures. For the CS structure, we set off‐diagonal elements $\omega_{rr^{\prime }}=\rho_{1}$ for $r\neq r^{\prime }$. For the AR(1) structure, we set $\omega_{rr^{\prime }}=\rho_{1}^{\left|t_{r}-t_{r^{\prime }}|/(t_{T}-t_{1}\right)}$. For the between‐subject correlation matrix (Φ), we specified $\Phi =11^{\prime }\rho_{3}+I(\rho_{2}-\rho_{3})$, where $\rho_{2}$ corresponds to the traditional ICC and we set the between‐subject between‐period correlation $\rho_{3}=\rho_{2}/2$. We explored four combinations of $R=\left\{\rho_{1},\rho_{2},\rho_{3}\right\}$:

$$\begin{array}{ll} & R_{1}=\{0.30,0.05,0.025\};\\ & R_{2}=\{0.30,0.03,0.015\};\\ & R_{3}=\{0.15,0.05,0.025\};\\ & R_{4}=\{0.15,0.03,0.015\}. \end{array}$$

For T=6, we considered various missing scenarios with four sets of marginal observational probabilities:

$$\begin{array}{ll} & \delta_{1}=(1.00,1.00,1.00,1.00,1.00,1.00);\\ & \delta_{2}=(1.00,0.97,0.94,0.90,0.75,0.60);\\ & \delta_{3}=(1.00,0.92,0.84,0.76,0.68,0.60);\\ & \delta_{4}=(1.00,0.85,0.70,0.66,0.63,0.60). \end{array}$$

Here, $\delta_{1}$ corresponds to the scenario of complete data, while $\delta_{2}-\delta_{4}$ represent different trends in the occurrence of missing data, but with the same dropout rate (40%) at the end of the study. We also consider three missing data patterns: IM, MM, and MIX with weight w=0.5, each leading to different joint observational probabilities. For each combination of design parameters, we calculated the required number of clusters. The empirical power and type I error rate were then evaluated using the following algorithm:

1. Calculate the required number of clusters (n) for a given design configuration using Formula (10). If n is odd, randomly allocate the clusters as (n+1)/2 and (n−1)/2 between the two treatment groups.
2. Generate cluster sizes $J_{i}$ (i=1,⋯,n) from the assumed discrete uniform distribution.
3. For each cluster, generate observations ($y_{i}$) from a multivariate normal distribution based on $\beta_{4}=\beta_{40}$ and other model parameters ($p,\beta_{1},\beta_{2},\beta_{3},\sigma^{2},\Omega ,\Phi$).
4. Generate missing data according to the assumed missing pattern and observational probabilities (δ).
5. Obtain the GEE estimate ${\hat{\beta }}_{4}$ using Formula (4) and its variance ${\hat{\sigma }}_{4}^{2}/n$ through $\sum_{n}=A_{n}^{-1}V_{n}A_{n}^{-1}$ using Formulas (7) and (8). If $\left|\sqrt{n}{\hat{\beta }}_{4}/{\hat{\sigma }}_{4}\right|>z_{1-\alpha /2}$, set the rejection indicator D=1; otherwise, set D=0.
6. Repeat Steps 2‐5 L=5,000 times to obtain $D_{l}$ for l=1,…,L. Calculate the empirical power as $\sum_{l=1}^{L}D_{l}/L$.

**FIGURE 1:**
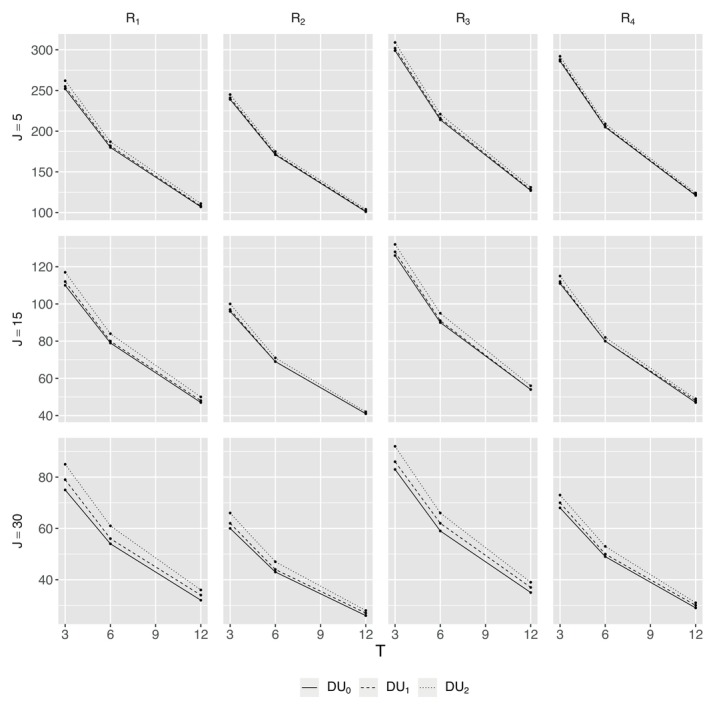
Required number of clusters for CRTLMs with complete observations and fixed ($DU_{0}$)/varying cluster sizes (${\text{DU}}_{1}$ and ${\text{DU}}_{2}$).

The empirical type I error can be obtained using the same algorithm except for setting $\beta_{4}=0$ in Step 3. Table 2 and Figure 1 (solid lines under ${\text{DU}}_{0}$) present the simulation results for CRTLMs with complete observations and fixed cluster size under various design configurations. Each cell in Table 2 shows the required number of clusters, along with the empirical power and type I error. We observe that the required number of clusters is negatively associated with cluster size (J), meaning that larger cluster sizes are associated with a smaller number of required clusters. Comparing the required number of clusters under different correlation parameter settings reveals additional insights. For instance, stronger longitudinal correlation ($\rho_{1}$) is associated with smaller sample size requirement ($R_{1}$ versus $R_{3}$), while stronger between‐subject correlations ($\rho_{2}$ and $\rho_{3}$) are associated with larger sample size requirement ($R_{1}$ versus $R_{2}$). As illustrated in the first row of Table 2, the required number of clusters increases from 252 to 299 with decreased longitudinal correlation from 0.3 to 0.15, while decreases from 252 to 239 with decreased between‐subject correlations.

**TABLE 2:** Required number of clusters (empirical power, empirical type I error) for CRTLMs with complete observations and fixed cluster sizes.

Correlation structure	J	T	$R_{1}$	$R_{2}$	$R_{3}$	$R_{4}$
z‐based						
CS	5	3	252 (0.8102, 0.0530)	239 (0.8136, 0.0554)	299 (0.8006, 0.0490)	286 (0.8016, 0.0516)
		6	180 (0.8040, 0.0518)	171 (0.8086, 0.0532)	214 (0.8076, 0.0518)	205 (0.7998, 0.0518)
		12	107 (0.8028, 0.0528)	101 (0.8026, 0.0578)	127 (0.7976, 0.0546)	121 (0.8088, 0.0570)
	15	3	110 (0.8202, 0.0524)	96 (0.8086, 0.0560)	126 (0.7982, 0.0546)	111 (0.8026, 0.0558)
		6	79 (0.8084, 0.0548)	69 (0.8094, 0.0604)	90 (0.8044, 0.0522)	80 (0.8102, 0.0530)
		12	47 (0.8140, 0.0618)	41 (0.8146, 0.0610)	54 (0.8162, 0.0572)	47 (0.8060, 0.0608)
	30	3	75 (0.8098, 0.0520)	60 (0.8042, 0.0594)	83 (0.8062, 0.0598)	68 (0.8164, 0.0544)
		6	54 (0.8222, 0.0572)	43 (0.8018, 0.0660)	59 (0.8098, 0.0598)	49 (0.8078, 0.0562)
		12	32 (0.8250, 0.0666)	26 (0.8328, 0.0730)	35 (0.8216, 0.0604)	29 (0.8330, 0.0696)
AR(1)	5	3	252 (0.8048, 0.0502)	239 (0.8008, 0.0580)	299 (0.8018, 0.0522)	286 (0.8048, 0.0484)
		6	259 (0.8042, 0.0494)	250 (0.7988, 0.0492)	307 (0.7972, 0.0534)	298 (0.7996, 0.0436)
		12	268 (0.7968, 0.0562)	263 (0.7984, 0.0504)	323 (0.8098, 0.0582)	317 (0.7922, 0.0574)
	15	3	110 (0.8088, 0.0576)	96 (0.8036, 0.0618)	126 (0.8068, 0.0478)	111 (0.8034, 0.0484)
		6	105 (0.8048, 0.0536)	95 (0.8038, 0.0572)	122 (0.8008, 0.0550)	111 (0.8040, 0.0550)
		12	101 (0.7996, 0.0610)	95 (0.8136, 0.0568)	119 (0.7980, 0.0568)	113 (0.8072, 0.0548)
	30	3	75 (0.8094, 0.0642)	60 (0.8232, 0.0570)	83 (0.8164, 0.0526)	68 (0.8220, 0.0614)
		6	67 (0.8112, 0.0606)	56 (0.8104, 0.0570)	75 (0.8086, 0.0604)	64 (0.8088, 0.0580)
		12	59 (0.8152, 0.0594)	53 (0.8190, 0.0592)	68 (0.8056, 0.0596)	62 (0.8086, 0.0590)
t‐based						
CS	5	3	252 (0.8072, 0.0516)	239 (0.8116, 0.0536)	299 (0.7992, 0.0484)	286 (0.7988, 0.0494)
		6	180 (0.7994, 0.0512)	171 (0.8050, 0.0520)	214 (0.8052, 0.0492)	205 (0.7966, 0.0514)
		12	107 (0.7984, 0.0514)	101 (0.7950, 0.0534)	127 (0.7922, 0.0516)	121 (0.8018, 0.0544)
	15	3	110 (0.8124, 0.0496)	96 (0.8034, 0.0530)	126 (0.7930, 0.0530)	111 (0.7960, 0.0534)
		6	79 (0.7974, 0.0490)	69 (0.7966, 0.0566)	90 (0.7954, 0.0482)	80 (0.8044, 0.0500)
		12	47 (0.8012, 0.0534)	41 (0.7962, 0.0542)	54 (0.8034, 0.0520)	47 (0.7922, 0.0558)
	30	3	75 (0.8008, 0.0486)	60 (0.7958, 0.0532)	83 (0.7966, 0.0558)	68 (0.8070, 0.0506)
		6	54 (0.8072, 0.0530)	43 (0.7848, 0.0574)	59 (0.7990, 0.0544)	49 (0.7940, 0.0490)
		12	32 (0.8026, 0.0572)	26 (0.8040, 0.0578)	35 (0.7966, 0.0496)	29 (0.8072, 0.0576)
AR(1)	5	3	252 (0.8014, 0.0496)	239 (0.7984, 0.0570)	299 (0.7996, 0.0508)	286 (0.8022, 0.0476)
		6	259 (0.8020, 0.0482)	250 (0.7970, 0.0488)	307 (0.7962, 0.0526)	298 (0.7978, 0.0430)
		12	268 (0.7934, 0.0558)	263 (0.7952, 0.0494)	323 (0.8088, 0.0570)	317 (0.7902, 0.0562)
	15	3	110 (0.8036, 0.0560)	96 (0.7954, 0.0584)	126 (0.8022, 0.0464)	111 (0.7972, 0.0456)
		6	105 (0.7978, 0.0504)	95 (0.7976, 0.0540)	122 (0.7956, 0.0530)	111 (0.7982, 0.0524)
		12	101 (0.7950, 0.0562)	95 (0.8080, 0.0534)	119 (0.7930, 0.0546)	113 (0.8006, 0.0516)
	30	3	75 (0.8008, 0.0594)	60 (0.8122, 0.0514)	83 (0.8092, 0.0500)	68 (0.8118, 0.0550)
		6	67 (0.8006, 0.0566)	56 (0.7964, 0.0512)	75 (0.7998, 0.0554)	64 (0.7994, 0.0528)
		12	59 (0.8034, 0.0528)	53 (0.8080, 0.0538)	68 (0.7970, 0.0534)	62 (0.7966, 0.0548)

**FIGURE 2:**
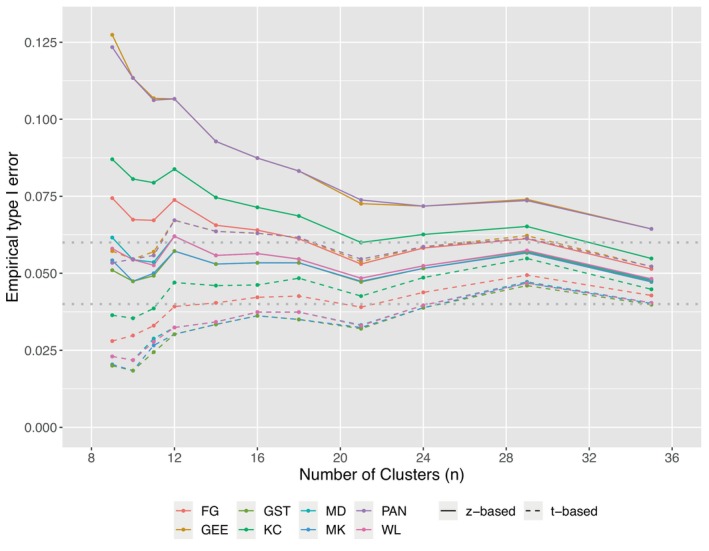
Empirical type I error under various GEE variance estimator adjustment methods combined with normal distribution (z‐based) or t distribution (t‐based) for statistical inference.

It is noteworthy that the empirical type I errors reported in Table 2 are slightly inflated, reaching up to 0.073. This is a well‐known issue in GEE inference for cluster randomized studies, resulting from an underestimation of the variance of the treatment effect under relatively smaller numbers of clusters. We found that using the t distribution for statistical inference provides a practical solution, which has been widely discussed and utilized [21, 22]. Specifically, in Step 5 of the simulation algorithm, we replaced the rejection threshold $z_{1-\alpha /2}$ with $t_{1-\alpha /2,n-4}$, which is the 100(1−α/2)th percentile of the t distribution with (n−4) degrees of freedom. The second part of Table 2 (t‐based) shows the empirical powers and type I errors using rejection threshold $t_{1-\alpha /2,n-4}$. Empirical powers and type I errors under this approach are much closer to their nominal levels. For studies with a relatively smaller number of clusters, we conducted additional simulations to evaluate the performance of various GEE variance estimator adjustment methods when combined with the normal distribution (z‐based) or t distribution (t‐based) for statistical inference. These methods are denoted by FG [23], GST [24], MD [25], PAN [26], KC [22], MK [27], and WL [28]. By fixing the randomization probability (p=0.5), variance ($\sigma^{2}=1$), number of time points (T=6), fixed cluster size (J=30), and correlation ($R=R_{1}$), we determined the required number of clusters (n) using Formula (6) to achieve the desired power of 1−γ=0.8 at a two‐sided significance level of α=0.05. This calculation was performed for various intervention effects ($\beta_{4}=0.05$ to 0.1 in increments of 0.005), yielding a required cluster count ranging from 9 to 35. Figure 2 illustrates the performance of these adjustment methods combined with the z‐based or t‐based approach under the CS correlation structure. The performance under AR(1) is similar and hence omitted. “GEE” in the figure represents the GEE method without adjustment. Overall, as the number of clusters increases, all adjustment methods converge to the nominal level of 0.05, albeit at different rates. The GST and MK methods demonstrate the best performance, maintaining type I error rates closest to 0.05. The WL and MD methods exhibit slightly higher type I error rates than GST and MK. Additionally, GEE and PAN combined with a t‐based rejection threshold show slight inflation for n<20, whereas KC and FG methods with a t‐based rejection threshold are conservative for n<12. The other adjustment methods tend to be either overly conservative or overly optimistic. In summary, for studies with fewer than 50 clusters, adjustment is often necessary to reduce bias for the estimated variance of the treatment effect. When the number of clusters exceeds 30, the t‐based method is a practical choice due to its straightforward implementation and lower computational burden compared to other adjustment methods. However, for fewer than 30 clusters, the GST and MK methods perform better in controlling the type I error. Notably, in the remainder of this paper, the simulation results presented use the t‐based rejection threshold.

We further investigated the effects of varying cluster sizes on sample size requirements for studies with complete observations. Table 3 and Figure 1 present our examination under CS correlation structure across three discrete uniform (DU) distributions for each cluster size, including one with no variability (fixed cluster size, denoted by ${\text{DU}}_{0}$), moderate variability (denoted by ${\text{DU}}_{1}$), and large variability (denoted by ${\text{DU}}_{2}$). Results under the AR(1) structure were similar and are therefore not shown. The findings highlight two main points:

1. greater variability in cluster size leads to a larger required number of clusters, and
2. by properly accounting for variability in cluster size, the proposed sample size method maintains powers and type I errors close to their nominal levels. This is a significant practical advantage, as a constant cluster size is rare in practice. The sample size Formula (10) only requires the specification of the mean and variance of $J_{i}$'s, rather than the exact distribution. This reduces the burden on practitioners in CRTLM design.

**TABLE 3:** Required number of clusters (empirical power, empirical type I error) for CRTLMs with complete observations and varying cluster sizes.

J	T	DU	$R_{1}$	$R_{2}$	$R_{3}$	$R_{4}$
5	3	[5,5]	252 (0.8072, 0.0516)	239 (0.7972, 0.0510)	299 (0.7962, 0.0516)	286 (0.8006, 0.0472)
		[3, 7]	255 (0.8018, 0.0488)	241 (0.7848, 0.0472)	302 (0.7966, 0.0530)	288 (0.8010, 0.0548)
		[1, 9]	262 (0.7932, 0.0530)	245 (0.8016, 0.0538)	309 (0.8008, 0.0512)	292 (0.7960, 0.0522)
	6	[5,5]	180 (0.8002, 0.0552)	171 (0.7942, 0.0490)	214 (0.8076, 0.0574)	205 (0.8034, 0.0506)
		[3, 7]	182 (0.7942, 0.0550)	172 (0.7910, 0.0526)	216 (0.7920, 0.0518)	206 (0.7864, 0.0506)
		[1, 9]	187 (0.7960, 0.0512)	175 (0.8116, 0.0478)	221 (0.7978, 0.0524)	209 (0.7986, 0.0504)
	12	[5,5]	107 (0.8018, 0.0536)	101 (0.7942, 0.0564)	127 (0.7996, 0.0496)	121 (0.7978, 0.0556)
		[3, 7]	108 (0.7984, 0.0538)	102 (0.7974, 0.0544)	128 (0.7960, 0.0482)	122 (0.7960, 0.0512)
		[1, 9]	111 (0.8050, 0.0536)	104 (0.8026, 0.0522)	131 (0.7904, 0.0534)	124 (0.7922, 0.0540)
15	3	[15,15]	110 (0.7976, 0.0482)	96 (0.8066, 0.0554)	126 (0.7850, 0.0512)	111 (0.7994, 0.0484)
		[10, 20]	112 (0.8028, 0.0546)	97 (0.8010, 0.0450)	128 (0.8028, 0.0484)	112 (0.7918, 0.0514)
		[5, 25]	117 (0.7972, 0.0562)	100 (0.8010, 0.0552)	132 (0.7920, 0.0570)	115 (0.7994, 0.0556)
	6	[15,15]	79 (0.8090, 0.0540)	69 (0.8190, 0.0578)	90 (0.8026, 0.0592)	80 (0.7896, 0.0556)
		[10, 20]	80 (0.8026, 0.0556)	69 (0.7996, 0.0526)	91 (0.7912, 0.0494)	80 (0.7940, 0.0498)
		[5, 25]	84 (0.8014, 0.0522)	71 (0.7958, 0.0612)	95 (0.7928, 0.0538)	82 (0.8044, 0.0522)
	12	[15,15]	47 (0.7972, 0.0582)	41 (0.7920, 0.0576)	54 (0.8034, 0.0580)	47 (0.7890, 0.0538)
		[10, 20]	48 (0.8022, 0.0542)	41 (0.7880, 0.0556)	54 (0.7926, 0.0516)	48 (0.7998, 0.0566)
		[5, 25]	50 (0.7968, 0.0494)	42 (0.7930, 0.0510)	56 (0.7914, 0.0600)	49 (0.7938, 0.0592)
30	3	[30,30]	75 (0.8004, 0.0510)	60 (0.8024, 0.0558)	83 (0.7996, 0.0558)	68 (0.7950, 0.0536)
		[15, 45]	79 (0.7922, 0.0586)	62 (0.8024, 0.0594)	86 (0.8010, 0.0538)	70 (0.7952, 0.0604)
		[5, 55]	85 (0.8048, 0.0544)	66 (0.8058, 0.0602)	92 (0.7994, 0.0500)	73 (0.7972, 0.0564)
	6	[30,30]	54 (0.8014, 0.0510)	43 (0.8004, 0.0498)	59 (0.7950, 0.0458)	49 (0.7956, 0.0524)
		[15, 45]	56 (0.7962, 0.0548)	44 (0.7946, 0.0562)	62 (0.8024, 0.0556)	50 (0.7902, 0.0540)
		[5, 55]	61 (0.7980, 0.0592)	47 (0.8054, 0.0592)	66 (0.7970, 0.0566)	53 (0.8008, 0.0566)
	12	[30,30]	32 (0.7930, 0.0582)	26 (0.7958, 0.0570)	35 (0.7884, 0.0568)	29 (0.8030, 0.0576)
		[15, 45]	34 (0.8172, 0.0548)	27 (0.8046, 0.0618)	37 (0.7988, 0.0556)	30 (0.7964, 0.0570)
		[5, 55]	36 (0.8046, 0.0602)	28 (0.7972, 0.0690)	39 (0.7904, 0.0574)	31 (0.7880, 0.0620)

**FIGURE 3:**
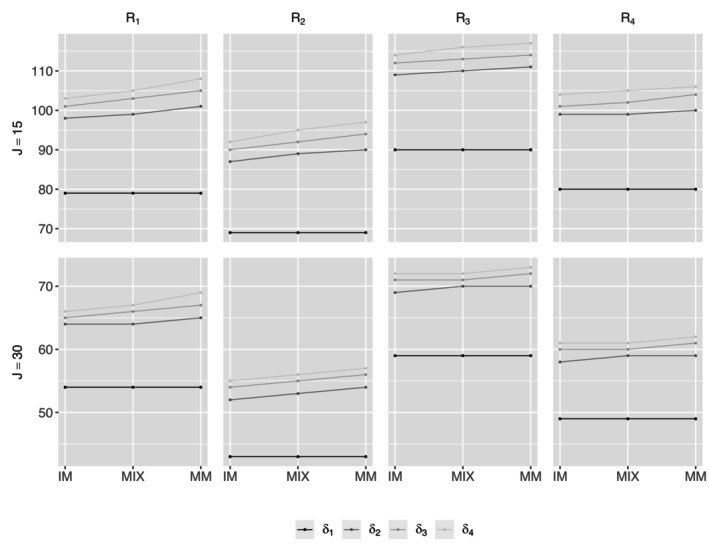
Required number of clusters for CRTLMs with incomplete observations and fixed cluster size.

On the other hand, in Table 4 and Figure 3 we investigated the impact of different missing patterns and observational probabilities on sample size requirement when cluster size is fixed. First, CRTLMs with missing data require more clusters to compensate for the loss of power. This is evident as the missing data scenarios always lead to a larger required number of clusters, as illustrated by the complete observations ($\delta_{1}$) versus missing data ($\delta_{2}-\delta_{4}$) in Figure 3. Second, the required number of clusters increases sequentially from $\delta_{2}$ to $\delta_{4}$, which corresponds to growing proportions of missing data, despite having the same dropout rate at the end of the study. Third, among the three missing patterns considered, the MM pattern requires the largest sample size due to the high concentration of missing data among a few subjects, leading to significant information loss. The IM pattern, conversely, results in the smallest sample size, while the MIX pattern falls in between. Finally, it is worth noting that the empirical powers and type I errors are well‐maintained across different missing patterns and observational probabilities, as presented in Table 4.

**TABLE 4:** Required number of clusters (empirical power, empirical type I error) for CRTLMs with incomplete observations and fixed cluster sizes.

J	Missing					
	Pattern	δ	$R_{1}$	$R_{2}$	$R_{3}$	$R_{4}$
15	IM	$\delta_{1}$	79 (0.8080, 0.0550)	69 (0.7996, 0.0538)	90 (0.7988, 0.0506)	80 (0.7992, 0.0560)
		$\delta_{2}$	98 (0.7954, 0.0506)	87 (0.7984, 0.0472)	109 (0.8014, 0.0550)	99 (0.8084, 0.0512)
		$\delta_{3}$	101 (0.8024, 0.0592)	90 (0.8010, 0.0506)	112 (0.7984, 0.0552)	101 (0.7962, 0.0552)
		$\delta_{4}$	103 (0.8132, 0.0556)	92 (0.8008, 0.0560)	114 (0.8106, 0.0508)	104 (0.8128, 0.0500)
	MM	$\delta_{2}$	101 (0.8098, 0.0518)	90 (0.8198, 0.0486)	111 (0.8208, 0.0554)	100 (0.8164, 0.0488)
		$\delta_{3}$	105 (0.8148, 0.0546)	94 (0.8224, 0.0502)	114 (0.8160, 0.0528)	104 (0.8252, 0.0536)
		$\delta_{4}$	108 (0.8184, 0.0498)	97 (0.8232, 0.0504)	117 (0.8154, 0.0454)	106 (0.8118, 0.0498)
	MIX	$\delta_{2}$	99 (0.7982, 0.0540)	89 (0.7952, 0.0474)	110 (0.7986, 0.0530)	99 (0.7960, 0.0548)
		$\delta_{3}$	103 (0.8058, 0.0524)	92 (0.8060, 0.0558)	113 (0.8078, 0.0528)	102 (0.8066, 0.0520)
		$\delta_{4}$	105 (0.8092, 0.0486)	95 (0.8084, 0.0598)	116 (0.8088, 0.0544)	105 (0.8072, 0.0458)
30	IM	$\delta_{1}$	54 (0.8100, 0.0504)	43 (0.8002, 0.0534)	59 (0.7978, 0.0582)	49 (0.7980, 0.0554)
		$\delta_{2}$	64 (0.8042, 0.0502)	52 (0.7946, 0.0516)	69 (0.7924, 0.0590)	58 (0.8002, 0.0534)
		$\delta_{3}$	65 (0.7964, 0.0584)	54 (0.8004, 0.0498)	71 (0.8034, 0.0542)	60 (0.8086, 0.0472)
		$\delta_{4}$	66 (0.8068, 0.0532)	55 (0.8038, 0.0548)	72 (0.7968, 0.0558)	61 (0.8056, 0.0556)
	MM	$\delta_{2}$	65 (0.8064, 0.0506)	54 (0.8062, 0.0550)	70 (0.8020, 0.0512)	59 (0.8072, 0.0524)
		$\delta_{3}$	67 (0.8232, 0.0530)	56 (0.8124, 0.0496)	72 (0.8128, 0.0524)	61 (0.8140, 0.0530)
		$\delta_{4}$	69 (0.8186, 0.0436)	57 (0.8168, 0.0526)	73 (0.8160, 0.0520)	62 (0.8060, 0.0572)
	MIX	$\delta_{2}$	64 (0.7934, 0.0520)	53 (0.7986, 0.0524)	70 (0.7996, 0.0510)	59 (0.8074, 0.0526)
		$\delta_{3}$	66 (0.8118, 0.0542)	55 (0.8038, 0.0516)	71 (0.8076, 0.0512)	60 (0.8082, 0.0562)
		$\delta_{4}$	67 (0.8034, 0.0552)	56 (0.8088, 0.0520)	72 (0.8044, 0.0548)	61 (0.8072, 0.0530)

**FIGURE 4:**
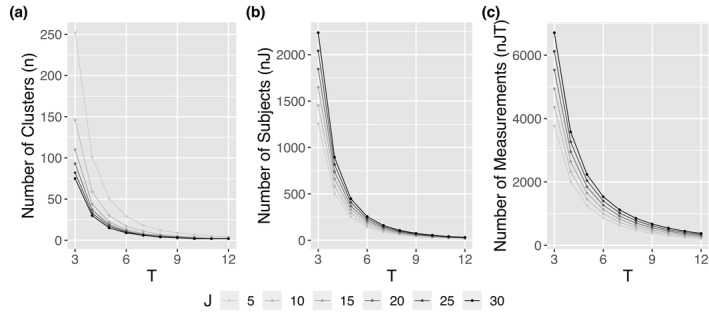
Relationship between the required number of clusters, total number of subjects, and total number of measurements with cluster size and the number of time points.

We further used the closed‐form sample size Formula (6) to investigate the relationship between the required number of clusters, total number of subjects, and total number of measurements with cluster size and the number of time points. By fixing the randomization probability (p=0.5), variance ($\sigma^{2}=1$), intervention effect ($\beta_{4}=0.1$), and correlations ($R=R_{1}$), we calculated the required number of clusters (n), total number of subjects (nJ), and total number of measurements (nJT) needed to achieve a desired power of 1−γ=0.8 at a two‐sided significance level of α=0.05. Figure 4 illustrates this relationship under the CS correlation structure. The pattern under AR(1) is similar and hence omitted. As shown in Figure 4a, when the cluster size (J) is fixed, increasing the number of measurements per subject (T) leads to a decrease in the number of clusters. Vertically, a larger cluster size (J) results in fewer clusters for a given T. Moreover, the effect of increasing T or J to reduce the number of clusters is greater when their initial values are smaller. For example, when J is fixed, increasing T from 3 to 4 is more effective in reducing the required clusters than increasing it from 4 to 5. Likewise, when T is fixed, expanding J from 5 to 10 is more effective than increasing it from 10 to 15. As shown in Figure 4b, when J is fixed, we observe the same trend as in Figure 4a: as T increases, the total number of subjects decreases. This is expected, as the total number of subjects is given by nJ, which depends only on n when J is fixed. However, vertically a different pattern emerges in which the total number of subjects (nJ) increases with cluster size (J). This occurs because while an increase in J leads to a decrease in n, the reduction in n is not sufficient to offset the increase in J. As shown in Figure 4c, as T increases, the total number of measurements (nJT) decreases for a given cluster size (J). This happens because the reduction in n, caused by the increase in T, has a greater impact on nJT than the increase in T. When T is fixed, we see the same trend as in Figure 4b: as the cluster size increases, the total number of measurements increases. This is expected because the total number of measurements is given by nJT, which depends only on n and J when T is fixed. In summary, to achieve the desired power, either increasing the number of time points for collecting measurements or increasing the cluster size will reduce the required number of clusters. Specifically, for studies with a fixed cluster size, increasing the number of time points will reduce the number of clusters, subjects, and measurements. In contrast, for studies with a fixed number of time points, increasing the cluster size may reduce the required number of clusters but increase the total number of subjects and measurements. An additional cost‐effectiveness analysis that considers the relative costs between adding a cluster, enrolling an additional patient, or taking additional measurement would inform the selection of the optimal design.

**FIGURE 5:**
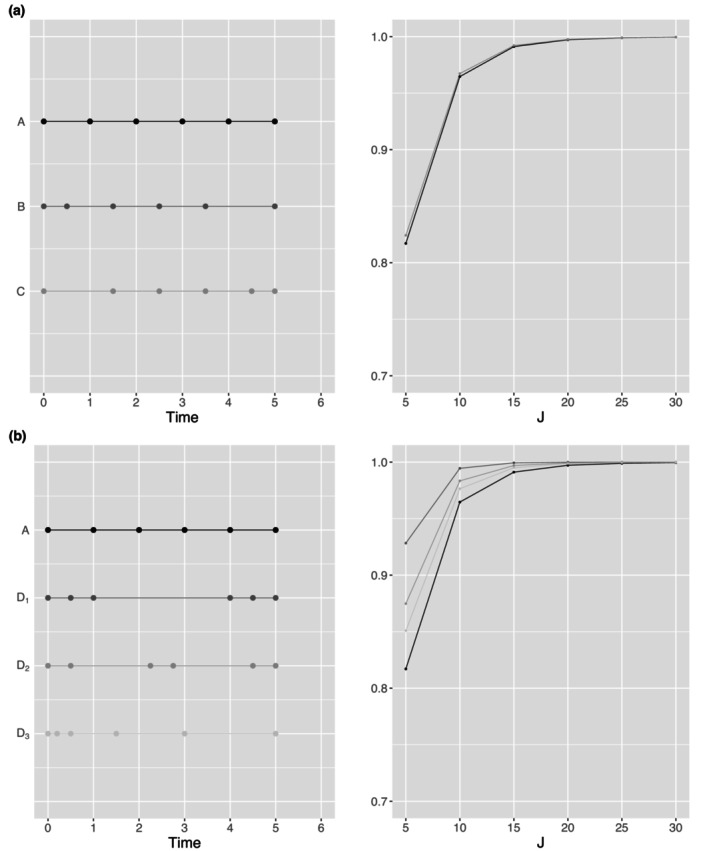
Impact of different measurement time patterns on statistical power.

**FIGURE 6:**
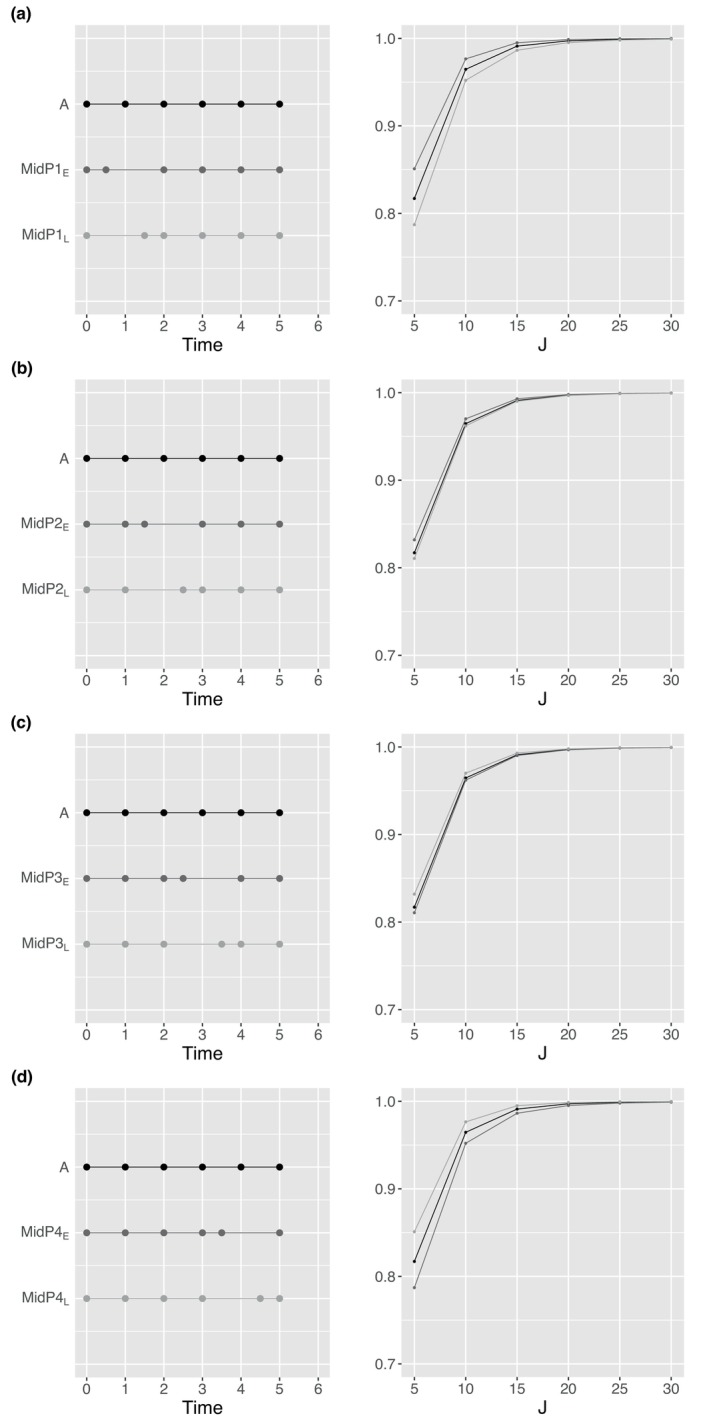
Impact of varying single measurement time point on statistical power.

In the studies above, we assumed evenly spaced time points for the longitudinal measurements. In practice, studies may require unevenly spaced time points due to clinical or logistical constraints, or subjects may not be able to provide measurements at prespecified times. Hence, we investigated the impact of different measurement time patterns on statistical power. By fixing the randomization probability (p=0.5), variance ($\sigma^{2}=1$), intervention effect ($\beta_{4}=0.1$), number of clusters (n=30), number of time points (T=6), and correlations ($R=R_{1}$ under CS), we calculated the achieved power using Formula (11) at a two‐sided significance level of α=0.05 for CRTLMs with a fixed cluster size and complete observations. Suppose subjects deviate from their planned schedule, as shown in Figure 5a. Pattern A illustrates the planned time points with even spacing ($t_{r}=r-1$ for r=1,⋯,T). Patterns B and C represent extreme cases where measurements are collected either earlier or later than planned for all time points except the first and last, given the same study duration. Figure 5a shows that B and C share the same power with overlapping lines due to the symmetric property that mirrored measurement time patterns will yield the same power. Moreover, Patterns B and C have slightly higher power compared to Pattern A, which led us to compare the power between the evenly spaced time pattern (A) and the more unevenly spaced time patterns ($D_{1}-D_{3}$) in Figure 5b. Specifically, Pattern $D_{1}$ has measurement times clustered at both the beginning and the end, Pattern $D_{2}$ has measurements clustered at the beginning, middle, and end, while Pattern $D_{3}$ features highly unbalanced timing, with more early measurements and fewer later ones. It shows that patterns with clustered time points near the beginning and end of the study are more efficient for slope estimation, resulting in higher power for slope comparisons. In addition, we investigated the impact of varying a single measurement time point on power, excluding the beginning and ending time points. As shown in Figure 6, we varied a single measurement time point earlier or later than its planned time. For example, two patterns representing variations in the first middle measurement time point are denoted as ${\text{MidP1}}_{E}$ (earlier) and ${\text{MidP1}}_{L}$ (later). Shifting time points that are originally closer to the beginning or end has a greater impact on power compared to moving time points that are originally near the middle ((a,d) vs. (b,c)). Furthermore, moving a time point closer to the beginning or end results in higher power than shifting it toward the middle (${\text{MidP1}}_{E}$ vs. ${\text{MidP1}}_{L}$ in (a) and (d)).

## 4 Example

We apply the proposed method to a CRTLM. Suppose we are designing a CRTLM to compare longitudinal trends in depression measurements between the usual care and intervention groups to investigate the effectiveness of the intervention [29]. Clinics are randomly assigned to either a control or intervention group, and patients within each clinic are repeatedly assessed for changes in depression symptoms using the 17‐Item Hamilton Rating Scale for Depression. Assessments are scheduled at baseline, 3, 6, 9, 12, and 15 weeks. The primary goal is to test whether depression symptoms decline more rapidly over the 15‐week period among patients treated at clinics assigned to the intervention group compared to those receiving usual care. The maximum number of measurements for each subject is T=6, although there are numerous missing values at later measurement times. We set $t_{r}=$ 0, 3, 6, 9, 12, and 15. At baseline, we assume a Hamilton depression score of 19.6 with a standard deviation of 6.4 in both intervention and control groups [29]. We expect the score to decline to 11 in the intervention group and 14 in the usual care group, indicating a standardized difference of −0.09 in slope between the two groups. With p=0.5 and a cluster size of J=10, we assume correlation values of R={0.15,0.05,0.025}. Based on the pilot data, if an AR(1) decay longitudinal correlation structure is appropriate, achieving 80% power at a two‐sided significance level of 0.05 requires 31 clinics. When accounting for observational probabilities δ=(1.00,0.85,0.70,0.66,0.63,0.60), the required number of clinics increases to 37 under the IM pattern and 41 under the MM pattern. If cluster size variability follows a DU distribution with a range of [5,15], the required number of clinics rises to 38 for the IM pattern and 42 for the MM pattern. Alternatively, if the CS longitudinal correlation structure is appropriate, 22 clinics are needed to achieve 80% power. Under the same observational probabilities, the requirement increases to 29 clinics for the IM pattern and 30 for the MM pattern. When cluster size variability follows a DU distribution with a range of [5,15], 30 clinics are required for both patterns after rounding.

## 5 Conclusions

In this study, we propose a closed‐form formula for calculating the required number of clusters for CRTLMs. Our method provides a comprehensive and flexible solution for sample size determination in CRTLMs, addressing practical design challenges often encountered in pragmatic trials, such as unbalanced randomization between treatment groups and varying cluster sizes. It also accommodates different correlation structures via two correlation matrices and accounts for missing data at the subject level by incorporating specifications for time‐dependent observational probabilities and various missing patterns. By adjusting the number of clusters to compensate for information loss due to missing data, the method ensures adequate power and reliable statistical inferences.

We developed our method within the GEE framework using an independent working correlation structure, which is often recommended for several reasons. First, it provides consistent parameter estimates regardless of the true correlation structure, ensuring robustness [18]. Second, it simplifies the estimation process and avoids the complexities associated with more structured correlation models, which require estimating nuisance parameters without a closed‐form sample size solution and may lead to convergence issues [17]. Third, the efficiency loss is generally minimal, particularly when the true correlations are weak or the sample size is large, making it a practical choice in many scenarios [30, 31, 32]. Finally, GEE with an independent working correlation structure is less sensitive to missing data assumptions compared to more structured correlation models, making it more suitable for datasets with irregular or unbalanced observations [33]. However, extending to a general working correlation structure would require specifying joint missing data probabilities, further complicating the sample size formula.

Across a wide range of design configurations, our simulations show that the proposed method achieves empirical power and type I error close to their nominal levels. The simulation results also suggest that the values and structures of correlation significantly impact the number of clusters required. In practice, a pilot study is recommended if reliable information on correlation parameters and structures is unavailable. Furthermore, it is important to note the potential issue of inflated type I errors for the GEE method under limited numbers of clusters. We demonstrate that using decision thresholds based on the t distribution instead of the normal distribution performs better in terms of maintaining power and type I error close to their nominal levels. Compared to other adjustment methods, this approach is easier to implement in practice. For a relatively small number of clusters, other adjustment methods, such as GST [24] or MK [27], may be necessary to effectively control the inflated Type I error.

In summary, we propose a practical closed‐form method for calculating sample size in CRTLMs using the GEE approach. The method accommodates unbalanced randomization, arbitrary correlation structures, missing data, and varying cluster sizes, ensuring accurate sample size estimations and maintaining desired power. This approach simplifies the design of CRTLMs, making it especially useful for researchers who need robust, adaptable solutions for designing complex cluster randomized studies with longitudinal measurements and missing data.

## Funding

This work was supported by the National Institutes of Health (Grant Nos. P30 CA142543 and 1ULTR003163).

## Disclosure

The authors have nothing to report.

## Conflicts of Interest

The authors declare no conflicts of interest.

## Supporting information

Data S1. Supporting Information.

## Data Availability

The authors have nothing to report.

## Appendix A Derivation of Formulas for Variances

The following derivation aims to establish Formula (9) for $\sigma_{4}^{2}$ under scenarios with incomplete observations. Formula (5) for $\sigma_{4}^{2}$ for complete observations can be obtained straightforwardly by treating all observation indicators $m_{ijr}$ as 1 in the derivation presented below.

For a CRTLM with incomplete observations, we define the marginal observational probability $\delta_{r}=P(m_{ijr}=1)$ for r=1,…,T, and the joint observational probability $\delta_{rr^{\prime }}=P(m_{ijr}m_{ijr^{\prime }}=1)$. Then we have

$$\begin{array}{ll} A_{n} & =n^{-1}\sum_{i=1}^{n}\sum_{j=1}^{J_{i}}\sum_{r=1}^{T}Z_{ijr}^{\prime }Z_{ijr}m_{ijr}\\ & =n^{-1}\sum_{i=1}^{n}\sum_{j=1}^{J_{i}}\sum_{r=1}^{T}{\left[1,u_{i}-p,t_{r},(u_{i}-p)t_{r}\right]}^{\prime }\left[1,u_{i}-p,t_{r},(u_{i}-p)t_{r}\right]m_{ijr}\\ & =n^{-1}\sum_{i=1}^{n}\sum_{j=1}^{J_{i}}\sum_{r=1}^{T}m_{ijr}\left[\begin{array}{cccc} 1 & u_{i}-p & t_{r} & \left(u_{i}-p\right)t_{r}\\ u_{i}-p & {\left(u_{i}-p\right)}^{2} & \left(u_{i}-p\right)t_{r} & {\left(u_{i}-p\right)}^{2}t_{r}\\ t_{r} & \left(u_{i}-p\right)t_{r} & t_{r}^{2} & \left(u_{i}-p\right)t_{r}^{2}\\ \left(u_{i}-p\right)t_{r} & {\left(u_{i}-p\right)}^{2}t_{r} & \left(u_{i}-p\right)t_{r}^{2} & {\left(u_{i}-p\right)}^{2}t_{r}^{2} \end{array}\right]. \end{array}$$

As n→∞, $A_{n}$ approaches

$$A=J\mu_{m0}\left[\begin{array}{cccc} 1 & 0 & \mu_{m1} & 0\\ 0 & v_{p}^{2} & 0 & v_{p}^{2}\mu_{m1}\\ \mu_{m1} & 0 & \mu_{m2} & 0\\ 0 & v_{p}^{2}\mu_{m1} & 0 & v_{p}^{2}\mu_{m2} \end{array}\right]$$

with the facts of $n^{-1}\sum_{i=1}^{n}\sum_{j=1}^{J_{i}}\sum_{r=1}^{T}m_{ijr}\to J\sum_{r=1}^{T}\delta_{r}$, $n^{-1}\sum_{i=1}^{n}\left(u_{i}-p\right)\to 0$, $n^{-1}\sum_{i=1}^{n}\sum_{j=1}^{J_{i}}\sum_{r=1}^{T}m_{ijr}t_{r}\to J\sum_{r=1}^{T}\delta_{r}t_{r}$, $n^{-1}\sum_{i=1}^{n}{\left(u_{i}-p\right)}^{2}\to p(1-p)$, and $n^{-1}\sum_{i=1}^{n}\sum_{j=1}^{J_{i}}\sum_{r=1}^{T}m_{ijr}t_{r}^{2}\to J\sum_{r=1}^{T}\delta_{r}t_{r}^{2}$, where $\mu_{m0}=\sum_{r=1}^{T}\delta_{r}$, $\mu_{m1}=\sum_{r=1}^{T}\delta_{r}t_{r}/\mu_{m0}$, $\mu_{m2}=\sum_{r=1}^{T}\delta_{r}t_{r}^{2}/\mu_{m0}$, and $v_{p}^{2}=p(1-p)$. Since A is a block diagonal‐like matrix with interlacing zero patterns, the inverse of A is

$$A^{-1}=\frac{1}{J\mu_{m0}v_{p}^{2}v_{mt}^{2}}\left[\begin{array}{cccc} v_{p}^{2}\mu_{m2} & 0 & -v_{p}^{2}\mu_{m1} & 0\\ 0 & \mu_{m2} & 0 & -\mu_{m1}\\ -\;v_{p}^{2}\mu_{m1} & 0 & v_{p}^{2} & 0\\ 0 & -\mu_{m1} & 0 & 1 \end{array}\right],$$

where $v_{mt}^{2}=\mu_{m2}-\mu_{m1}^{2}$.

On the other hand, we have

where ${\hat{e}}_{ij}=y_{ij}-Z_{i}\hat{b}$, $m_{ij}={\left(m_{ij1},\ldots ,m_{ijT}\right)}^{\prime }$, and ∘ is the Hadamard product. As n→∞, $V_{n}$ approaches

$$V=\sigma^{2}J^{2}\left[\begin{array}{cccc} 1_{T}^{\prime }Q_{m}1_{T} & 0 & 1_{T}^{\prime }Q_{m}t & 0\\ 0 & v_{p}^{2}1_{T}^{\prime }Q_{m}1_{T} & 0 & v_{p}^{2}1_{T}^{\prime }Q_{m}t\\ t^{\prime }Q_{m}1_{T} & 0 & t^{\prime }Q_{m}t & 0\\ 0 & v_{p}^{2}t^{\prime }Q_{m}1_{T} & 0 & v_{p}^{2}t^{\prime }Q_{m}t \end{array}\right]$$

with the facts of $Z_{i}=\left(1_{T},(u_{i}-p)1_{T},t,(u_{i}-p)t\right)$, $n^{-1}\sum_{i=1}^{n}2\sum_{j=1}^{J_{i}-1}\sum_{j^{\prime }=j+1}^{J_{i}}1=n^{-1}\sum_{i=1}^{n}J_{i}(J_{i}-1)\to J^{2}+\sigma_{J}^{2}-J=J^{2}\left(CV_{J}^{2}+1-\frac{1}{J}\right)$, $n^{-1}\sum_{i=1}^{n}\sum_{j=1}^{J_{i}}\left({\hat{e}}_{ij}\circ m_{ij}\right){\left({\hat{e}}_{ij}\circ m_{ij}\right)}^{\prime }\to J\Omega \circ \Delta$, and $n^{-1}\sum_{i=1}^{n}\left({\hat{e}}_{ij}\circ m_{ij}\right){\left({\hat{e}}_{ij^{\prime }}\circ m_{ij^{\prime }}\right)}^{\prime }\to \text{diag}(\delta )\Phi \text{diag}(\delta )$, where $Q_{m}=\frac{1}{J}\Omega \circ \Delta +(CV_{J}^{2}+1-\frac{1}{J})\text{diag}(\delta )\Phi \text{diag}(\delta )$. Here diag(δ) is a diagonal matrix with diagonal elements being $\delta ={\left(\delta_{1},\ldots ,\delta_{T}\right)}^{\prime }$ and Δ is a matrix with the (r,r)th element being $\delta_{r}$ and the $\left(r,r^{\prime }\right)$th element being $\delta_{rr^{\prime }}$ ($r\neq r^{\prime }$). Then

$$\begin{array}{cc} \sum & =A^{-1}VA^{-1}\\ & =\frac{\sigma^{2}J^{2}}{{\left(J\mu_{m0}v_{p}^{2}v_{mt}^{2}\right)}^{2}}\left[\begin{array}{cccc} v_{p}^{2}\mu_{m2} & 0 & -v_{p}^{2}\mu_{m1} & 0\\ 0 & \mu_{m2} & 0 & -\mu_{m1}\\ -\;v_{p}^{2}\mu_{m1} & 0 & v_{p}^{2} & 0\\ 0 & -\mu_{m1} & 0 & 1 \end{array}\right]\\ & \;\left[\begin{array}{cccc} 1_{T}^{\prime }Q_{m}1_{T} & 0 & 1_{T}^{\prime }Q_{m}t & 0\\ 0 & v_{p}^{2}1_{T}^{\prime }Q_{m}1_{T} & 0 & v_{p}^{2}1_{T}^{\prime }Q_{m}t\\ t^{\prime }Q_{m}1_{T} & 0 & t^{\prime }Q_{m}t & 0\\ 0 & v_{p}^{2}t^{\prime }Q_{m}1_{T} & 0 & v_{p}^{2}t^{\prime }Q_{m}t \end{array}\right]\\ & \;\left[\begin{array}{cccc} v_{p}^{2}\mu_{m2} & 0 & -v_{p}^{2}\mu_{m1} & 0\\ 0 & \mu_{m2} & 0 & -\mu_{m1}\\ -\;v_{p}^{2}\mu_{m1} & 0 & v_{p}^{2} & 0\\ 0 & -\mu_{m1} & 0 & 1 \end{array}\right]. \end{array}$$

Consequently, the (4,4)th element in $\sum =A^{-1}VA^{-1}$ is

$$\sigma_{2}^{2}=\frac{\sigma^{2}}{\mu_{m0}^{2}v_{p}^{2}v_{mt}^{4}}{\left(\mu_{m1}1_{T}-t\right)}^{\prime }Q_{m}\left(\mu_{m1}1_{T}-t\right).$$
